# Epigenetic modification of hypothalamic neuropeptides and metabolic hormone receptors in metabolic health

**DOI:** 10.3389/fendo.2025.1645474

**Published:** 2025-09-17

**Authors:** Busayo Oladun, Smita Mall, Min-Hyun Kim

**Affiliations:** College of Health Solutions, Arizona State University, Phoenix, AZ, United States

**Keywords:** hypothalamus, epigenetics, neuropeptides, metabolism, obesity, metabolic hormone receptors, energy homeostasis

## Abstract

The hypothalamus plays a central role in regulating metabolism by integrating neuropeptide signaling with environmental cues to maintain energy homeostasis. Adverse environmental factors, such as obesogenic diet, undernutrition, stress, and sedentary lifestyles, can disrupt the normal regulation of key hypothalamic neuropeptides and metabolic hormone receptors through epigenetic mechanisms, including DNA methylation, histone modifications, and microRNA regulation. These epigenetic alterations are not merely transient; they can be heritable and may influence metabolic health across generations, highlighting the critical need to understand the underlying epigenetic mechanisms. In this review, we provide a comprehensive overview of how environmental factors shape the epigenetic landscape of hypothalamic neuropeptides (pre-opiomelanocortin, neuropeptide Y, and agouti-related peptide) and metabolic hormone receptors (leptin receptor and insulin receptor), thereby modulating their expression and contributing to long-term metabolic outcomes. A better understanding of environment-epigenome interactions holds promise for the development of innovative therapeutic strategies to combat obesity and metabolic disorders.

## 1 Introduction

The brain is a critical organ in regulating metabolism, with several anatomical regions pivotal to maintaining energy homeostasis (1, 2). Among these, the hypothalamus emerges as a central regulator, coordinating many physiological processes in the body, including the control of appetite, energy metabolism, stress response, thermoregulation, circadian rhythms, the autonomic nervous system, the regulation of body fluid, control of endocrine systems, and emotional and behavioral responses (3). Key hypothalamic nuclei involved in energy regulation include the arcuate nucleus (ARC), ventromedial hypothalamus (VMH), dorsomedial hypothalamus (DMH), and paraventricular nucleus (PVN) (4–10). These structures work synergistically to integrate internal and external cues, maintaining energy balance through neuropeptides and metabolic receptors.

Hypothalamic neuropeptides such as neuropeptide Y (NPY), agouti-related peptide (AgRP), pro-opiomelanocortin (POMC), and cocaine- and amphetamine-regulated transcript (CART) play contrasting roles in energy homeostasis. NPY and AGRP stimulate appetite, promote food intake, and reduce energy expenditure (11, 12), whereas POMC and CART inhibit food intake, promote satiety, and increase energy expenditure (13). Additionally, receptors for metabolic hormones like leptin and insulin contribute to metabolic function in the hypothalamus. Dysregulation of leptin and insulin signaling has been linked to the development of metabolic disorders (14, 15).

Environmental factors, including diet, stress, and physical activity, modulate hypothalamic neuropeptides and metabolic hormone receptors expression, and dysfunction in their expression has been linked to metabolic disorders (16–19). Overnutrition, particularly energy-dense diets, and chronic stress have been implicated in epigenetic modifications leading to the development and progression of metabolic diseases, particularly obesity (20). Such changes are often prolonged, persisting across an individual’s lifespan and potentially being transmitted across generations (21, 22). This has led to a growing interest in understanding the role of epigenetics as a key regulator of gene-environment interactions in the development of metabolic disorders. Thus, exploring the epigenetic underpinnings of these structures and their activities will improve our understanding of the anomalies associated with metabolic health and provide potential remedies.

Epigenetic mechanisms, including DNA methylation, histone modifications, and non-coding RNAs such as microRNAs (miRNAs), have been identified as key regulators of hypothalamic neuropeptides and metabolic hormone receptors (23). These mechanisms influence cellular transcriptional potentials, thereby modulating phenotypes associated with energy regulation (24). For instance, stress and diet-induced epigenetic changes have been implicated in altered expression of neuropeptides like POMC, AgRP, and NPY, and receptors such as the leptin receptor (LepRb) and the insulin receptor (InsR) (25–28). Additionally, the CART and endocannabinoid system (ECS) represent other epigenetically modulated pathways with implications on energy homeostasis. Recent advancements in understanding the complex interactions between epigenetic modifications and neuropeptide gene expression have increased the potential for therapeutic interventions. This review considers an overview of the current understanding of the epigenetic regulation of hypothalamic neuropeptides and some metabolic hormone receptors and their functional roles, followed by an in-depth analysis of how environmental factors influence their responses or expression. This will deepen our understanding and hold promise to advance personalized therapeutic strategies in preventing and treating metabolic diseases.

### 1.1 DNA methylation

DNA methylation is a key epigenetic mechanism involving the addition of a methyl group to the fifth carbon of cytosine (one of the four nitrogen bases in DNA, the others being adenine, thymine, and guanine) to form 5-methylcytosine (29). This modification primarily occurs at cytosines adjacent to guanine nucleotides, known as CpG sites, although evidence suggests methylation can also occur at non-CpG sites (30). CpG islands, regions with a high density of CpG sites, are often located near gene promoters, especially the housekeeping genes (31). These regions are crucial in gene regulation, as they regulate chromatin structure and transcription binding factors. Methylation of the CpG islands typically results in gene silencing, whereas unmethylation or demethylation promotes gene expression (32). Environmental factors, such as diet and stress, have been implicated in altering DNA methylation patterns, linking this mechanism to metabolic disorders like diabetes and obesity (33). Given that the thymus and the brain are the two most highly methylated human tissues (34), the hypothalamus, being a critical regulator of energy homeostasis, emerges as an important area for exploring the crosstalk between DNA methylation and metabolic regulation.

### 1.2 Histone modifications

The DNA is condensed in a complex structure known as chromatin, which is made up of histones H2A, H2B, H3, and H4 (35, 36). The degree of chromatin compaction determines the accessibility of DNA to transcription, repair, and replication machinery. Opening up the chromatin structure facilitates gene activation, while more tightly packed chromatin suppresses gene expression. Histone modifications, such as acetylation, methylation, phosphorylation, sumoylation, deamination, and proline isomerization, play a crucial role in gene expression by influencing the access of transcription factors to DNA (37). Acetylation and phosphorylation promote transcription by loosening the chromatin and enhancing transcription factor access (38), whereas sumoylation, deamination, and proline isomerization generally lead to gene silencing. Depending on the specific lysine residues involved, methylation and ubiquitination exhibit dual roles, either activating or repressing genes (39, 40). Two primary mechanisms underlie these histone modifications: (a) disrupting nucleosome-nucleosome interactions to open chromatin and (b) recruiting non-histone proteins that further modify chromatin through enzymatic activities (39). These dynamic modifications are critical for understanding the regulation of metabolic pathways, especially in the hypothalamus, which governs energy balance and appetite control.

### 1.3 MicroRNAs

miRNAs are small non-coding RNA molecules of 21 – 26 nucleotides that regulate gene expression by binding to the 3^’^ untranslated region (3^’^UTR) of message-encoding RNAs (41–43). This binding can either degrade the resulting messenger RNA (mRNA) or inhibit its translation, depending on the level of complementarity between the miRNA and its target mRNA. Perfect complementarity results in cleavages and degradation, whereas imperfect complementarity initiates mRNA silencing by specific mechanisms, including translational repression, sequestration in cytoplasmic processing bodies, and/or slicer-dependent mRNA degradation (44). The outcome of miRNA processes is gene repression or silencing, which has been implicated in many diseases, especially metabolic disorders (45–47). Dysregulated miRNAs influence key hypothalamic neuropeptides involved in appetite and energy balance, contributing to conditions like obesity. Thus, we shall review the metabolic effects of hypothalamic neuropeptides and receptors in light of these epigenetic changes.

### 1.4 Pro-opiomelanocortin

In the brain, POMC, a 30-kDa prohormone, is highly expressed in the arcuate nucleus of the hypothalamus, pituitary gland, and brain stem (48, 49). Depending on the expression levels of prohormone convertases, POMC gives rise to various active peptides and hormones, such as melanocyte-stimulating hormones (MSH) (α-MSH, β-MSH, and γ-MSH), adrenocorticotropic hormone (ACTH), corticotropin-like intermediate lobe peptide (CLIP), Lipotropin (γ-lipotropin and β-lipotropin), and met-enkephalin (50, 51). As an anorexigenic peptide, POMC suppresses appetite and increases energy expenditure, leading to weight loss primarily through the melanocortin signaling pathway, an important mechanism for maintaining energy balance (52). Beyond regulating metabolism, these products also regulate stress response, the immune system, and sexual functions (53–55).

The pivotal role of POMC was illustrated by Krude et al. (56), who described two patients with congenital absence of the *Pomc* gene and its derived peptides. One patient carried two nonsense mutations in exon 3, which led to losing key *Pomc*-derived peptides, including ACTH, α-MSH, and β-MSH. The second patient had a homozygous mutation in the 5’-untranslated region of *Pomc*, impairing the proper translational initiation of the POMC protein. Both patients presented with early-onset obesity due to hyperphagia linked to impaired melanocortin signaling in the hypothalamus, along with hypercortisolemia and distinctive physical traits like pale skin and red hair due to reduced activation of MC1R in melanocytes (56). Further evidence by Farooqi et al. (57), demonstrated that loss of even one allele of *Pomc* predisposes individuals to obesity, with 11 out of 12 heterozygous patients being either overweight or obese (57). Animal studies also alluded to the role of *Pomc* in energy balance, as *Pomc*-deficient mice and zebrafish developed severe obesity, increased food intake, and insulin resistance (58–62). These findings emphasize that hypothalamic POMC is critical for regulating energy balance.

Various factors mediate the epigenetic modifications of *Pomc*, influencing the phenotypic expression and metabolic outcomes. For instance, a high-fat diet (HFD) has been shown to influence DNA methylation of the *Pomc* gene promoter, thus affecting its expression. In a study by Cifani et al. (63), rats fed an HFD (45% kcal fat) were classified as diet-induced obese or diet-resistant rats based on their weight gain. While there was no difference in the *Pomc* expression levels at the 5th week between diet-induced obese and diet-resistant rats, by the 21st week, *Pomc* levels were elevated in diet-resistant rats. Examination of the *Pomc* promoter revealed decreased DNA methylation at the CpG sites 1,2,6, and 7 in diet-resistant rats compared to diet-induced obese rats (63). Furthermore, rats fed an HFD (60% kcal fat) from post-weaning to adulthood exhibited DNA hypermethylation in specific regions of the *Pomc* promoter, leading to reduced *Pomc* expression and increased body weight when compared to the control (64). These findings indicate that HFD promotes DNA hypermethylation of the *Pomc* promoter, which decreases *Pomc* expression. This reduction disrupts energy balance and feeding behavior, potentially contributing to HFD-induced obesity.

Similar results were observed in human studies. However, due to the challenges associated with directly investigating epigenetic changes in the living human hypothalamus, researchers have explored the use of alternative approaches, such as postmortem hypothalamus. In a study using postmortem hypothalamic samples from obese and normal-weight individuals with no history of neurodegenerative disease or cancer, researchers performed a laser microdissection of MSH-positive neurons. They found that the *Pomc* methylation in these neurons positively correlated with the basal metabolic index of the individual (65). Specifically, obese individuals showed increased methylation at a CpG island at the intersection between *Pomc* intron and coding exon 3, compared to non-obese individuals, thereby suggesting that the *Pomc* hypermethylation in the MSH neurons may impair satiety signaling and promote obesity. Additionally, using leukocytes, one study examined the effects of an 8-week hypocaloric weight loss program designed to induce a 30% energy restriction (500 – 600 kcal/day) with a macronutrient composition of 55% carbohydrate, 15% protein, and 30% fat, on *Pomc* methylation in obese male subjects. The participants were categorized as either “weight regainers” or “non-regainers” based on weight changes observed 32 weeks after stopping dieting. Interestingly, the CpG sites 10 and 11 of *Pomc* showed higher methylation levels in the regainers than non-regainers at baseline (66). This finding suggests that the methylation status of the *Pomc* promoter region is critical to regulating weight gain and may provide a useful biomarker for potential early detection and differential diagnosis of the predisposition to regain dietary-induced weight loss.

Another factor that affects the methylation status of *Pomc* is undernutrition, which is culpable in anorexia nervosa. The peripheral blood mononuclear cells of healthy women, underweight patients, and weight-recovered patients with anorexia nervosa were examined for specific DNA methylation of the *Pomc* gene expression (67). The researchers found that *Pomc* expression was higher in underweight patients than in weight-recovered patients or healthy controls. Although there was no significant difference in the overall DNA methylation between the groups, specific associations were found between the DNA methylation of single CpG residues and the expression of *Pomc* mRNA (67). This suggests that changes in *Pomc* expression observed in patients with anorexia nervosa may be more closely related to nutritional deficiencies rather than the direct effects of DNA methylation patterns or modifications.

Other factors, like stress and gender, also influence the DNA methylation of *Pomc*. A study on the early life stress caused by 3 hours of daily separation of pups from their dams over 10 consecutive days reduced DNA methylation, increasing *Pomc* mRNA in the pituitary gland (68). This suggests that early stress exposure may upregulate *Pomc* mRNA, potentially influencing long-term energy homeostasis. However, how much stress exposure will be beneficial is still debatable, given that prolonged stress has long-term metabolic consequences. Sex-specific differences in the DNA methylation status of the *Pomc* gene have been reported in various species, highlighting the role of epigenetic regulation of hypothalamic neuropeptides in metabolism. For instance, a study that determined the sex differences in the DNA methylation patterns across eight sites of the *Pomc* promoter of a 3-week-old chicken observed different methylation patterns across the promoter. Female chickens displayed higher methylation levels across the *Pomc* promoter than males (69). This elevated methylation in females was associated with reduced *Pomc* gene expression, suggesting that sex-specific epigenetic modifications may contribute to differences in metabolic regulation between male and female chickens.

However, demethylating the *Pomc* promoter does not always produce lean or energy-regulated phenotypes. For example, the cafeteria diet (CAF), which is an experimental rodent diet model that provides up to 4.85 kcal/g, 49% of energy as fat, 7% as protein, and 44% as carbohydrate, has been shown to influence *Pomc* expression (70). Exposure to the CAF diet was found to reduce methylation at the *Pomc* promoter in rats after 11 weeks and 20 weeks of feeding; however, this demethylation of the *Pomc* promoter did not adequately counteract the increased food intake observed, suggesting that an additional orexigenic mechanism is responsible for the increase in body weight observed (71). Similarly, targeted demethylation of the *Pomc* promoter by using CRISPR-dCas9-TET1 in HFD (60% kcal fat) fed rats did not prevent weight gain. Methylation repression using CRISPR-dCas9-DNMT3a also failed to alter body weight significantly (72), suggesting that *Pomc* promoter methylation changes may result from weight gain during obesity development rather than being its direct cause.

Further studies explored how adverse environmental factors influence histone modifications at the *Pomc* enhancer region using chromatin immunoprecipitation (ChIP) analysis. In one study, pups fed a high-carbohydrate diet exhibited decreased histone acetylation of H3K9 (H3K9ac) at the *Pomc* promoter compared to mother-fed pups, thereby reducing *Pomc* expression and contributing to diet-induced obesity (73). Adolescent alcohol exposure in rats was shown to increase histone acetylation of H3K9/14 at the *Pomc* promoter, leading to elevated *Pomc* mRNA levels that continued into adulthood (74). However, the result is different for offspring born to parents who consumed alcohol. A study also showed that prenatal exposure to ethanol in rats led to the production of offspring with suppressed histone activation marks (H3K4me3) and increased repressive marks (H3K9me2), thereby reducing *Pomc* mRNA levels (75). The differences might be because prenatal exposure affects the early development of the brain during critical epigenetic reprogramming, whereas key brain structures like the hypothalamus are already developed before adolescent alcohol exposure. Nonetheless, it is clear that environmental factors such as diet and alcohol can influence histone marks and *Pomc* expression, affecting metabolic outcomes.

miRNAs play critical regulatory roles in gene expression, including modulating hypothalamic *Pomc* expression, which is pivotal in maintaining energy homeostasis. For example, in leptin-deficient (ob/ob) mice, several miRNAs, such as miR-383, miR-384-3p, and miR-488, were upregulated and have been shown to negatively regulate *Pomc* mRNA expression (76). The downregulation of *Pomc* leads to disruptions in energy balance and contributes to the development of obesity. Importantly, with the treatment of leptin in these animals, there was the restoration of *Pomc* mRNA levels, underscoring the dynamic regulation of *Pomc* by leptin-induced changes in miRNAs, positioning them as potential therapeutic targets for metabolic disorders (76). Further evidence that shows the involvement of miRNAs in metabolic regulation comes from a study on the effect of prenatal alcohol exposure to mice between postnatal day 2 and postnatal day 6, which is the equivalent of the third trimester in humans due to the similarity in brain development. It was observed that prenatal alcohol exposure upregulated miR-383 and miR-384 while the *Pomc* gene expression was reduced in the mediobasal hypothalamus at postnatal days 6 and 60, respectively (77). These findings emphasize the influence of environmental factors, such as diet and prenatal exposure, on miRNA-mediated *Pomc* regulation, revealing a critical pathway by which miRNAs may impact metabolic health and disease risk across the lifespan.

Moreover, miRNA-specific manipulations can influence *Pomc* expression in the brain, particularly the hypothalamus. For instance, the knockdown of miR-342 in mice increased *Pomc* expression in response to a high-fat, high-sucrose diet, leading to reduced food intake and reduced body weight, thus implicating miR-342 expression in the development and progression of obesity (78). Additionally, loss of miR-29a in *Pomc* neurons contributes to hyperphagia, decreased energy expenditure, and obesity in female mice (79). Suffice it to mention that miR-375 has been identified as a negative regulator of *Pomc* expression in the pituitary gland. Inhibition of miR-375 led to an approximately 40% increase in the *Pomc* expression levels, while upregulation of miR-375 decreased *Pomc* expression levels (80). Put together, these findings highlight the intricate regulatory network governed by miRNAs in the hypothalamus, particularly in the *Pomc* neurons, and targeting these miRNAs could be beneficial in managing obesity and its related comorbidities.

The transgenerational effect of diet and environmental factors on epigenetic regulation of DNA methylation of hypothalamic neuropeptides is gaining attention among researchers. The DNA methylation of the *Pomc* promoter can occur as early as embryonic development and at different cell states. One study examining the *Pomc* DNA methylation of human embryonic stem cells (hESCs) observed that naive cells showed decreased DNA methylation compared to primed H1 cells, even after differentiation (81). However, when capacitated cells were used for hypothalamic neuronal differentiation, there was a marked increase in *Pomc* DNA methylation at the progenitor stage and even in *Pomc*-expressing neurons. The researchers further identified a negative correlation between *Pomc* DNA methylation and *Pomc* gene expression at CpG 1 to 7 during human embryogenesis (81). This early occurrence of DNA methylation of the *Pomc* gene implies that metabolic disease risk could be programmed at the cellular level before birth. As a result, if the methylation patterns persist or are exacerbated postnatally by either diet or environmental factors, they could predispose individuals to metabolic diseases by disrupting energy balance and promoting obesity.

Neonatal DNA methylation is often associated with maternal gestational age and nutritional patterns during these periods (82). In one study, offspring of mice dams fed with a high-fat, high-sucrose diet during pregnancy exhibited increased *Pomc* mRNA expression and hypomethylation of *Pomc* promoter in the hypothalamus, contributing to metabolic dysfunction in adulthood (83). Also, mice dams exposed to ethanol consumption had offspring with increased DNA-methylation enzyme (DNMT1), causing an increase in *Pomc* gene methylation and a concurrent reduction in *Pomc* mRNA expression (75). These findings suggest that the parents’ nutritional status has the potential to alter the methylation status of *Pomc* in the offspring, which may impact the *Pomc* expression, causing altered regulation of food intake, energy expenditure, and glucose homeostasis in adulthood.

### 1.5 Agouti-related peptide

AgRP is a potent orexigenic neuropeptide that stimulates appetite and reduces energy expenditure, acting as an antagonist to melanocortin receptors and predominantly present in the arcuate nucleus (84–86). In mice, using designer receptors exclusively activated by designer drugs (DREADD) to stimulate *AgRP* neurons resulted in increased food intake, reduced energy expenditure, and increased adiposity. On the other hand, inhibition of *AgRP* neuronal activity reduced food intake, highlighting the critical role of AgRP in regulating feeding and energy balance (87). Also, when rats were exposed to a low-protein diet, they exhibited increased *AgRP* mRNA expression and enhanced food intake, whereas intracerebroventricular injection of amino acids decreased *AgRP* mRNA and suppressed food intake (88). When *AgRP* is knocked out in mice and Siberian hamsters, the animals exhibit a reduced feeding drive and reduced weight gain (89, 90). All of these findings show the pivotal role of AgRP in influencing energy balance.

It is noteworthy that the different parts of the hypothalamus do not respond the same way to factors initiating *AgRP* activation. For example, a study in rats found an increased *AgRP* expression in the PVN and VMH, and a reduction in the ARC, following a cafeteria diet (91). When rat dams were fed a restricted protein diet during gestation and lactation, their pups displayed reduced *AgRP* expression in the PVN, while there was no change in the dorsomedial and lateral hypothalamus among pups fed by (92).

DNA methylation of the AgRp promoter region is less studied because of the lack of CpG sites in the promoter region. Some researchers could not even examine the DNA methylation of the *AgRP* promoter because it does not contain any CpG sites in the 1200 nucleotides upstream of the transcription sites (91, 92). In one study where rat pups born to caloric-restricted dams showed an increased *AgRP* expression and a decrease in birth weight, the researchers found that three CpG sites were 75% to 96% methylated (93). Another study on maternal HFD exposure revealed sex-specific differences in *AgRP* promoter methylation; male offspring displayed increased methylation at four CpG sites associated with a reduction in *AgRP* expression when compared to female offspring (94). However, all these sites are positioned outside the CpG islands of the *AgRP* promoter region.

Histone modification of *AgRP* has been observed to play a crucial role in regulating feeding behavior in the hypothalamus by influencing its expression and involvement in energy homeostasis. In rats that experienced caloric restriction following HFD-induced obesity, reduced repressive histone 3 lysine 9 methylation (H3K9me2) at the promoter of *AgRP* led to increased *AgRP* expression and heightened hunger signaling, suggesting anxadaptive response to restore energy homeostasis (95). Additionally, male rat offspring nursed by high-fat fed (60% Kcal fat) dams and weaned to control diet (10% Kcal fat) had elevated *AgRP* expression at six months with a significant increase in lysine-specific histone demethylase 1 (LSD1) and a decrease in histone deacetylase 1 (HDAC1) highlighting the effects of maternal HFD on the hypothalamic energy regulation of their offspring (96).

Not many studies examined the regulatory roles of miRNAs in *AgRP* gene expression, likely due to the intricate feedback and redundant signaling mechanisms that control appetite and metabolism. In sheep, brain-derived neurotrophic factor (BDNF) infusion increases *AgRP* expression in the ARC with a concurrent increase in miRNA-33a-5p, miRNA-33 b-5p, and a reduction in miRNA-377-3p and miRNA-214-3p, suggesting the role of these miRNAs in the regulation of *AgRP* expression (97). Further research, however, is still required to examine how miRNAs influence *AgRP* gene expression in the hypothalamus, as this gene is crucial in energy homeostasis.

### 1.6 Neuropeptide Y

NPY is an orexigenic peptide that is abundantly present in the arcuate nucleus of the hypothalamus (98). It plays a key role in regulating feeding behavior by stimulating appetite and increasing hunger, leading to increased food consumption, fat accumulation, weight gain, and obesity (99). NPY exerts its effects by binding to its G protein-coupled receptors, primarily Y1R, which is a key receptor driving appetite, along with other receptors like Y2R, Y4R, Y5R, and Y6R (100). *Npy* and *AgRP* neurons are colocalized in the arcuate nucleus, providing synergistic effects (101). A study reported that mice deficient in *Npy* exhibited normal food intake and body weight under basal conditions, a phenomenon similarly observed in *AgRP* knockout mice (102). A knockout of *Npy* or its receptors rarely yields a marked phenotype because compensatory mechanisms, such as other appetite-regulating neuropeptides like *AgRP*, adjust to offset the deficiency (102).

Recent research has significantly advanced our understanding of how epigenetic modifications impact *Npy* mRNA and its promoter under different environmental influences. For example, a study employed the use of restriction enzymes to assess the DNA methylation status of the *Npy* promoter in rats fed a cafeteria diet (CAF) at different feeding periods (71). They found that there was a reduction in DNA methylation of the *Npy* promoter at 11 weeks (medium term) and 20 weeks (long term) but not 4 weeks (short term), leading to an increase in *Npy* mRNA contributing to overeating and weight gain (71). This finding indicates that the duration of environmental exposure significantly influences epigenetic modifications in neuropeptides like *Npy*, potentially explaining variations in metabolic phenotypes. Another study showed that diet-resistant rats exhibited DNA methylation at the 5^th^ CpG site of the *Npy* promoter region when compared to diet-induced obese rats (63). The increased methylation in diet-resistant rats corresponded with reduced *Npy* expression, which correlated with lower food intake and weight gain resistance compared to diet-induced obese rats, thus preventing the rats from gaining weight. Additionally, a clinical study examined the methylation status of the *Npy* promoter region in obese males undergoing an 8-week hypocaloric diet intervention and observed at 8 and 32 weeks after the intervention, using leukocytes. The weight regainers showed a significant decrease in the methylation levels of *Npy* CpG sites 4 and 8 compared to non-regainers, even at baseline (66). Apart from the fact that hypomethylation at these sites is associated with decreased *Npy* expression, this finding also suggests that the methylation status of the *Npy* promoter region may provide a useful biomarker for potential early detection and differential diagnosis of the predisposition to regain dietary-induced weight loss.

Furthermore, studies have also shown that there is a transgenerational effect of diet-induced epigenetic changes in *Npy* expression. For example, pups of dams fed a high-carbohydrate diet showed reduced DNA methylation at the *Npy* promoter, leading to increased *Npy* expression (73). These epigenetic alterations were associated with hyperphagia, leading to increased weight gain and, subsequently, obesity. This finding highlights that parental epigenetic marks can persist through epigenetic reprogramming during conception, thereby influencing the susceptibility to metabolic diseases in adulthood.

Histone modification at the *Npy* promoter has also been studied. Rats fed a high-carbohydrate diet showed increased acetylation of H3K9 at the *Npy* promoter, which was linked to elevated *Npy* expression, contributing to obesity. The same diet did not significantly affect H3K9 methylation levels, suggesting that histone acetylation may play a more prominent role in diet-induced obesity (73). In another study where sex differences in the mice hypothalamic neurons are explored, the researchers found that H3K27me3 at the *Npy* promoter was higher in males than in females, indicating a more suppressive effect in males. siRNA-mediated knockdown of Kdm6a, a histone demethylase that specifically targets H3K27, resulted in increased H3K27me3 at the *Npy* promoter in females but not in males (103). This suggests that different types of histone modifications, such as methylation and acetylation, have distinct roles in regulating the target gene expression of *Npy* and may be sex-specific.

Besides, stress-related factors have been shown to be associated with epigenetic changes in *Npy*. For example, traumatic brain injury was associated with a significant reduction in H3K9ac levels at the *Npy* promoter in the arcuate nucleus, reducing food intake (104). Likewise, phenyl butyric acid, an endoplasmic reticulum stress inhibitor as well as a histone deacetylase inhibitor, has been shown to increase *Npy* mRNA levels by increasing H3K9/14ac at the promoter of *Npy*, further implicating a potential role of histone acetylation in the development of obesity (105). These findings suggest that stress or injury-induced changes to histone marks can suppress *Npy* expression, thereby reducing appetite and potentially altering energy balance. Moreover, pharmacologically targeting histone acetylation can modulate *Npy* expression and influence feeding behavior and weight gain.

miRNAs are also involved in regulating *Npy* expression. For example, the downregulation of miR-103/107 in mice is associated with reduced *Npy* mRNA expression, thereby affecting energy homeostasis (106). Mice lacking miR-342 showed a significant reduction in the total number of activated *Npy* in response to a high-fat, high-sugar diet in mice (78). In humans, miR-4713 and miR-452 were shown to be associated with increased *Npy*1r among obese children when assessed by miRwalk2.0, a tool for miRNA target prediction (107). These findings suggest that interventions targeting specific miRNAs might enhance our understanding of metabolic control to combat obesity through the modulation of *Npy* signaling.

### 1.7 Leptin receptor

The leptin receptor (LepR) belongs to the class 1 cytokine receptor family (108, 109). It exists in two primary isoforms: short forms and long forms (110). While the short form has limited functionality, the long form (LepRb) plays a crucial role in leptin signaling (111). LepRb is predominantly expressed in the central nervous system, especially in the hypothalamus, a region central to energy homeostasis. Circulating leptin, produced from the adipose tissue, mediates its effect by binding to LepRb in the brain, stimulating downstream signaling to regulate energy homeostasis. Studies have demonstrated that the dysfunction of LepRb or leptin signaling is involved in the development and progression of obesity. For instance, a mutation in the *LepRb* gene in humans resulted in weight gain and hyperphagia similar to those observed in leptin-deficient transgenic mice (112, 113). Deletion of *LepRb* in somatotrophs of the hypothalamus results in obesity (114), emphasizing the essential role of hypothalamic leptin receptors in maintaining energy balance.

The epigenetic modification of LepRb within the hypothalamus is critical to metabolism. One study in mice observed that a maternal HFD increased the DNA methylation levels of the *LepRb* promoter, increasing weight gain and reducing LepRb expression among the pups (115). Although specific CpG sites were not identified, higher global methylation levels were correlated with the decreased expression of LepRb. Several studies have failed to detect significant DNA methylation and found no significant DNA methylation of *LepRb* in the hypothalamus (116–118), potentially due to the unique epigenetic environment of this brain region. It is likely that leptin signaling may rely more on post-translational modifications or receptor sensitivity rather than DNA methylation changes.

Histone modification of leptin receptors in the hypothalamus has been shown to regulate metabolism. It was found that mice fed a high-fat diet upregulated slug, a transcription factor that recruits EZH2, which represses LepRb expression by increasing H3K27 dimethylation/trimethylation (H3K27me2/3) at the LepRb promoter in the hypothalamus (119), suggesting that HFD can epigenetically impact leptin receptor expression, thereby promoting and developing metabolic disorders.

miRNA also regulates LepRb expression. The inhibition of miR-200 in the hypothalamus was observed to increase the expression of LepRb, resulting in suppressed appetite, reduced food intake, and ultimately leading to reduced body weight (120), suggesting the potential roles of microRNAs in regulating *LepRb* mRNA. Further research on histone and miRNA modifications is needed to fully elucidate the impact of epigenetic changes on regulating LepRb to combat metabolic diseases.

### 1.8 Insulin receptor

In the brain, InsR is highly expressed in various brain regions, including the hypothalamus, olfactory bulb, cerebral cortex, cerebellum, and choroid plexus (121–123). InsR has two isoforms known as “A” and “B” (124). The “A” isoform is primarily found in the brain, while the “B” isoform is expressed in the liver, muscle, adipocytes, and kidney (125). Insulin, produced from the beta cells of the pancreas, crosses the blood-brain barrier to activate InsR in the brain. However, InsR’s role extends beyond glucose metabolism because only a fraction of glucose supplied to the neurons is insulin-dependent. In the hypothalamus, InsR, as well as their substrates, are vital for energy balance (126). In a transgenic mouse model where Insr-2 is knocked out, the female mice had an increase in food intake and body weight when compared to the control mice. No changes were observed in male mice (127). When a selective decrease in hypothalamic insulin receptor expression was performed using osmotic pumps to infuse oligodeoxynucleotide antisense, the rats became hyperphagic, had increased fat mass, and glucose intolerance (128, 129). These findings highlight the importance of hypothalamic insulin receptors in energy homeostasis and the need to examine how epigenetic modifications impact their functions.

Overfeeding or overnutrition has been shown to alter the DNA methylation of InsR. In a study that examined the effects of neonatal overfeeding on InsR in the hypothalamus, the researchers observed an increase in methylation of the CpG island of the insulin receptor promoter in over-nourished rats, resulting in rapid weight gain and the development of metabolic syndrome (130). Another study in rats showed that a maternal HFD led to hypermethylation of the hypothalamic InsR in male offspring but not female, leading to hyperleptinemia, hyperinsulinemia, impaired glucose tolerance, increased insulin resistance, and obesity (131). Collectively, these findings suggest early life overnutrition can induce sex -specific epigenetic modifications of hypothalamus *InsR*, thereby predisposing offspring to long-term metabolic dysregulation and obesity.

miRNAs have been observed to regulate InsR in the hypothalamus. In diabetic rats, miR-194-5p and miR-200a-3p are upregulated, leading to reduced InsR protein levels while Inhibition of these miRNAs restores InsR levels (132). Similarly, inhibition of miR-200 in the hypothalamus of db/db mice increased *InsR* expression, suppressed appetite, reduced food intake, and decreased body weight (120). These findings highlight the roles of hypothalamic miRNAs in regulating central insulin sensitivity and energy balance.

### 1.9 CART and endocannabinoid system

In addition to well-characterized neuropeptides such as NPY and POMC, several other hypothalamic regulators, including CART, and components of endocannabinoid systems, play a pivotal role in controlling energy homeostasis, feeding behaviors, and metabolic adaptation. Although their physiological significance is well established, the epigenetic mechanism governing their expression and activity in response to environmental cues remains incompletely understood.

CART is a key neuropeptide in the hypothalamus that plays a crucial role in regulating energy balance and feeding behavior. As an anorexigenic peptide, CART suppresses appetite and reduces food intake (133). Knockout studies have shown that deletion of *Cart* results in increased body weight and the development of obesity (134, 135), emphasizing its critical role in maintaining energy balance. Hypermethylation of the *Cart* promoter has been linked to reduced *Cart* mRNA expression in calorie-restricted rats, contributing to imbalanced energy intake and obesity (136). Although the epigenetic regulation of *Cart* remains underexplored, it has been suggested that many of its physiological effects may overlap with those of *Pomc*. This highlights the need for more studies on the epigenetic regulation of *Cart*, particularly in the context of metabolic adaptations and disease.

The endocannabinoid system, which consists of the cannabinoid receptor 1 (CB1R) and cannabinoid receptor 2 (CB2R), is an important mechanism involved in metabolism. Activation of CB1R in the ARC and VMH stimulates appetite and promotes energy storage, contributing to obesity, inflammation, and insulin resistance (137, 138). Mice lacking CB1R are resistant to diet-induced obesity, demonstrating the regulatory roles of endocannabinoid signaling in energy balance (139). Maternal HFD increases CB1R expression in the offspring and leads to sex-specific epigenetic modifications. For instance, female offspring of rats not affected by maternal HFD exhibited lower methylation at a CpG site within the CB1R intron but higher histone H3 acetylation in the promoter region. In contrast, male offspring exposed to maternal HFD showed increased histone acetylation (140). These findings suggest that diet-induced epigenetic modifications in CB1R may differ by sex and environmental exposures. Although CB2R has also been implicated in body weight control and glucose homeostasis, its epigenetic regulation within the hypothalamus remains unexplored.

## 2 Conclusion and future direction

**Figure 1:**
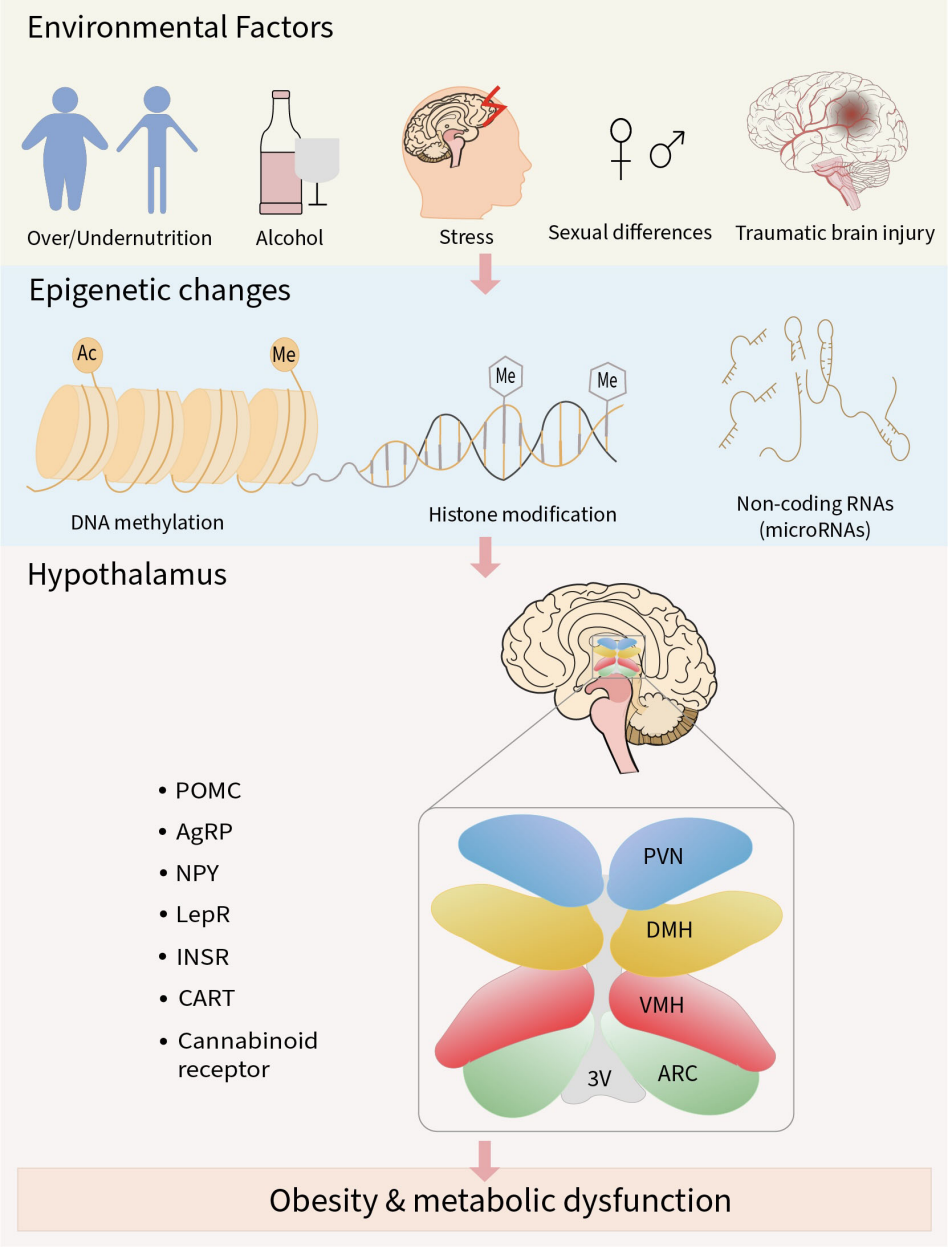
Schematic overview of the review. Adverse environmental factors can result in abnormal epigenetic modifications in neuropeptides and metabolic hormone receptors involved in the regulation of energy metabolism. Sustained exposure to these factors may lead to impaired metabolic signaling, contributing to the development of obesity and related metabolic disorders.

The hypothalamus is a complex neuronal network with notable cell populations equipped to maintain energy homeostasis by integrating hormonal, neural, and nutrient-related signals. It regulates both short-term appetite and long-term metabolic adaptation to prevent energy imbalance and the development of metabolic disorders such as obesity (141). Despite increasing evidence linking hypothalamic dysfunction to metabolic disease, the role of epigenetic regulation in this brain region remains underexplored, warranting a comprehensive review to synthesize current knowledge and identify future research gaps. In this review, we have discussed how adverse environmental factors such as malnutrition, alcohol consumption, stress, traumatic brain injury, and sex-specific differences can trigger epigenetic changes in the hypothalamus. Clinical and preclinical studies have demonstrated that epigenetic changes, including DNA methylation, histone modification, and regulation by non-coding RNA (miRNAs), can modify the expression of genes critical for energy balance, such as the neuropeptides (POMC, AgRP, NPY, CART) and metabolic hormone receptors (LepRb, InsR, cannabinoid receptors). Disruption of these signaling pathways within the hypothalamic nuclei (ARC, VMH, DMH, PVN) impairs the brain’s ability to integrate metabolic cues, leading to abnormal appetite regulation and reduced energy expenditure. Over time, these changes promote the development of obesity and related metabolic disorders (**Figure 1**).

Despite robust evidence from animal models, translating these mechanistic insights to humans remains challenging. Ethical and logistical constraints severely limit access to human hypothalamic tissue, hindering direct validation of animal-based findings. Potential solutions include the use of postmortem hypothalamic tissue repositories, the generation of human induced pluripotent stem cell (iPSC)-derived hypothalamic neurons, and the development of 3D brain organoids to model human-specific epigenetic regulation (142). Furthermore, non-invasive neuroimaging integrated with circulating biomarkers, such as miRNAs, may provide functional proxies for hypothalamic activity in living subjects. Beyond accessibility, the complexity of epigenetic regulation, being cell-type specific, dynamic, and shaped by multiple environmental inputs, requires advanced methodologies. Future research should incorporate single-cell epigenomic profiling, longitudinal study designs, and careful consideration of sex differences to achieve a more precise understanding of hypothalamic epigenetic mechanisms in metabolic diseases.

A deeper grasp of these mechanisms creates a foundation for novel therapeutic strategies targeting hypothalamic epigenetic dysregulation in metabolic disease. One promising strategy is miRNA modulation, such as miR-200 and miR-34a, which influence neuropeptide and hormone receptor expression involved in appetite control and energy homeostasis (120, 143–145). Therapeutic agents like miRNA mimics or antagomirs could normalize dysregulated pathways and restore metabolic balance. Another approach involves reprogramming histone modification states through drugs that activate or inhibit histone deacetylases(HDACs), histone methyltransferases (HMTs), or demethylases. Importantly, some HDAC inhibitors are already in clinical use or undergoing trials for cancer (146–149), offering opportunities for repurposing toward metabolic disorders. Finally, precision epigenetic editing tools, such as CRISPR-Cas9-based epigenetic editors, could allow locus-specific modulation of gene expression, directly correcting pathogenic chromatin states within the hypothalamic neurons. Collectively, these strategies rapidly define an emerging field, where mechanistic insight and technological innovation converge to combat obesity and metabolic syndrome at their epigenetic roots.

## References

[1] Harrold JA Dovey TM Blundell JE Halford JCG. CNS regulation of appetite. Neuropharmacology. (2012), 3–17. doi: 10.1016/j.neuropharm.2012.01.007, PMID: 22313528

[2] Grill HJ Schwartz MW Kaplan JM Foxhall JS Breininger J Baskin DG. Evidence that the caudal brainstem is a target for the inhibitory effect of leptin on food intake. Endocrinology. (2002) 143:239–46. doi: 10.1210/endo.143.1.8589, PMID: 11751615

[3] Saper CB Lowell BB. The hypothalamus. Curr Biol. (2014), R1111–6. doi: 10.1016/j.cub.2014.10.023, PMID: 25465326

[4] Sainsbury A Zhang L. Role of the arcuate nucleus of the hypothalamus in regulation of body weight during energy deficit. Mol Cell Endocrinology. (2010) 316:109–19. doi: 10.1016/j.mce.2009.09.025, PMID: 19822185

[5] Sutton AK Myers MG Olson D. The role of PVH circuits in leptin action and energy balance. Annu Rev Physiol. (2016), 207–21. doi: 10.1146/annurev-physiol-021115-105347, PMID: 26863324 PMC5087283

[6] Yang L Scott KA Hyun J Tamashiro KL Tray N Moran TH. Role of dorsomedial hypothalamic neuropeptide Y in modulating food intake and energy balance. J Neurosci. (2009) 29:179–90. doi: 10.1523/JNEUROSCI.4379-08.2009, PMID: 19129396 PMC2742174

[7] Gavini CK Jones WC Novak CM. Ventromedial hypothalamic melanocortin receptor activation: regulation of activity energy expenditure and skeletal muscle thermogenesis. J Physiol. (2016) 594:5285–301. doi: 10.1113/tjp.2016.594.issue-18, PMID: 27126579 PMC5023712

[8] Monda M Amaro IS De Luca B. The influence of exercise on energy balance changes induced by ventromedial hypothalamic lesion in the rat. Physiol Behav. (1993) 54(6):1057–61. doi: 10.1016/0031-9384(93)90324-9, PMID: 8295940

[9] Rui L. Brain regulation of energy balance and body weight. Rev Endocr Metab Disord. (2013) 14:387–407. doi: 10.1007/s11154-013-9261-9, PMID: 23990408 PMC3817823

[10] Rossi MA. Control of energy homeostasis by the lateral hypothalamic area. Trends Neurosciences. (2023), 738–49. doi: 10.1016/j.tins.2023.05.010, PMID: 37353461 PMC10524917

[11] Deem JD Faber CL Morton GJ. AgRP neurons: Regulators of feeding, energy expenditure, and behavior. FEBS J. (2022), 2362–81. doi: 10.1111/febs.v289.8, PMID: 34469623 PMC9040143

[12] Luo N Marcelin G Liu SM Schwartz G Chua S. Neuropeptide Y and Agouti-Related Peptide Mediate Complementary Functions of Hyperphagia and Reduced Energy Expenditure in Leptin Receptor Deficiency. Endocrinology. (2011) 152(3):883–9., PMID: 21285324 10.1210/en.2010-1135 PMC3040058

[13] Hill JW. Gene expression and the control of food intake by hypothalamic POMC/CART neurons. Open Neuroendocrinol J. (2010) 3:21–7., PMID: 28042349 PMC5201111

[14] Könner AC Brüning JC. Selective insulin and leptin resistance in metabolic disorders. Cell Metab. (2012), 144–52. doi: 10.1016/j.cmet.2012.07.004, PMID: 22883229

[15] Hosoi T Maffei M. Editorial: Leptin resistance in metabolic disorders: Possible mechanisms and treatments. Front Endocrinol. (2017). doi: 10.3389/fendo.2017.00300, PMID: 29163368 PMC5673631

[16] Carmo-Silva S Cavadas C. Hypothalamic dysfunction in obesity and metabolic disorders. Adv Neurobiol. (2017), 73–116. doi: 10.1007/978-3-319-63260-5_4, PMID: 28933062

[17] de Lima RMS dos Santos Bento LV di Marcello Valladão Lugon M Barauna VG Bittencourt AS Dalmaz C. Early life stress and the programming of eating behavior and anxiety: Sex-specific relationships with serotonergic activity and hypothalamic neuropeptides. Behav Brain Res. (2020) 379:112399. doi: 10.1016/j.bbr.2019.112399, PMID: 31790781

[18] Benite-Ribeiro SA Putt DA Santos JM. The effect of physical exercise on orexigenic and anorexigenic peptides and its role on long-term feeding control. Med Hypotheses. (2016) 93:30–3. doi: 10.1016/j.mehy.2016.05.005, PMID: 27372853

[19] Ziotopoulou M Mantzoros CS Hileman SM Flier JS. Differential expression of hypothalamic neuropeptides in the early phase of diet-induced obesity in mice. Am J Physiology-Endocrinology Metab. (2000) 279:E838–45. Available online at: http://www.ajpendo.orgE838., PMID: 11001766 10.1152/ajpendo.2000.279.4.E838

[20] Alegría-Torres JA Baccarelli A Bollati V. Epigenetics and lifestyle. Epigenomics. (2011) 3:267–77. doi: 10.2217/epi.11.22, PMID: 22122337 PMC3752894

[21] Vickers M. Early life nutrition, epigenetics and programming of later life disease. Nutrients. (2014) 6:2165–78. doi: 10.3390/nu6062165, PMID: 24892374 PMC4073141

[22] Sales VM Ferguson-Smith AC Patti ME. Epigenetic mechanisms of transmission of metabolic disease across generations. Cell Metab. (2017) 25:559–71. doi: 10.1016/j.cmet.2017.02.016, PMID: 28273478 PMC5404272

[23] Gibney ER Nolan CM. Epigenetics and gene expression. Heredity. (2010), 4–13. doi: 10.1038/hdy.2010.54, PMID: 20461105

[24] Holliday R. Epigenetics: A historical overview. Epigenetics. (2006) 1:76–80. doi: 10.4161/epi.1.2.2762, PMID: 17998809

[25] Mariano IR Yamada LA Soares Rabassi R Rissi Sabino VL Bataglini C Azevedo SCSF. Differential responses of liver and hypothalamus to the nutritional condition during lactation and adult life. Front Physiol. (2020) 11. doi: 10.3389/fphys.2020.00553, PMID: 32581843 PMC7291834

[26] Gołyszny M Obuchowicz E Zieliński M. Neuropeptides as regulators of the hypothalamus-pituitary-gonadal (HPG) axis activity and their putative roles in stress-induced fertility disorders. Neuropeptides. (2022). doi: 10.1016/j.npep.2021.102216, PMID: 34974357

[27] Marco A Kisliouk T Weller A Meiri N. High fat diet induces hypermethylation of the hypothalamic Pomc promoter and obesity in post-weaning rats. Psychoneuroendocrinology. (2013) 38:2844–53. doi: 10.1016/j.psyneuen.2013.07.011, PMID: 23958347

[28] Plagemann A Harder T Brunn M Harder A Roepke K Wittrock-Staar M. Hypothalamic proopiomelanocortin promoter methylation becomes altered by early overfeeding: An epigenetic model of obesity and the metabolic syndrome. J Physiol. (2009) 587:4963–76. doi: 10.1113/tjp.2009.587.issue-20, PMID: 19723777 PMC2770159

[29] Moore LD Le T Fan G. DNA methylation and its basic function. Neuropsychopharmacology. (2013), 23–38. doi: 10.1038/npp.2012.112, PMID: 22781841 PMC3521964

[30] Xie W Barr CL Kim A Yue F Lee AY Eubanks J. Base-resolution analyses of sequence and parent-of-origin dependent DNA methylation in the mouse genome. Cell. (2012) 148:816–31. doi: 10.1016/j.cell.2011.12.035, PMID: 22341451 PMC3343639

[31] Saxonov S Berg P Brutlag DL. A genome-wide analysis of CpG dinucleotides in the human genome distinguishes two distinct classes of promoters. Proceedings of the National Academy of Sciences. (2006) 103(5):1412–7. doi: 10.1073/pnas.0510310103, PMID: 16432200 PMC1345710

[32] Mohn F Weber M Rebhan M Roloff TC Richter J Stadler MB. Lineage-specific polycomb targets and *de novo* DNA methylation define restriction and potential of neuronal progenitors. Mol Cell. (2008) 30:755–66. doi: 10.1016/j.molcel.2008.05.007, PMID: 18514006

[33] Barres R Zierath JR. DNA methylation in metabolic disorders. Am J Clin Nutr. (2011) 93(4):897S–900S. doi: 10.3945/ajcn.110.001933, PMID: 21289222

[34] Ehrlich M A-Gama-Sosa M Huang L-H Midgett RM Kuo KC McCune RA. Amount and distribution of 5-methylcytosine in human DNA from different types of tissues or cells. Nucleic Acids Res. (1982) 10(8):2709–21. doi: 10.1093/nar/10.8.2709, PMID: 7079182 PMC320645

[35] Zhang Y Sun Z Jia J Du T Zhang N Tang Y. Overview of histone modification. (2021), 1–16. doi: 10.1007/978-981-15-8104-5_1, PMID: 33155134

[36] Roth SY Denu JM David Allis C. Histone acetyltransferases. Annu Rev Biochem. (2001) 70(1):81–120. doi: 10.1146/annurev.biochem.70.1.81, PMID: 11395403

[37] Zhao Y Garcia BA. Comprehensive catalog of currently documented histone modifications. Cold Spring Harb Perspect Biol. (2015) 7(9):a025064. doi: 10.1101/cshperspect.a025064, PMID: 26330523 PMC4563710

[38] Bannister AJ Kouzarides T. Regulation of chromatin by histone modifications. Cell Res. (2011), 381–95. doi: 10.1038/cr.2011.22, PMID: 21321607 PMC3193420

[39] Kouzarides T. Chromatin modifications and their function. Cell. (2007), 693–705. doi: 10.1016/j.cell.2007.02.005, PMID: 17320507

[40] Jaskelioff M Peterson CL. Chromatin and transcription: histones continue to make their marks. Nat Cell Biol. (2003) 5:395–9. doi: 10.1038/ncb0503-395, PMID: 12724776

[41] Lagos-Quintana M Rauhut R Lendeckel W Tuschl T. Identification of novel genes coding for small expressed RNAs. Science (1979). (2001) 294(5543):853–8. doi: 10.1126/science.1064921, PMID: 11679670

[42] Schneeberger M Gomez-Valadés AG Ramirez S Gomis R Claret M. Hypothalamic miRNAs: Emerging roles in energy balance control. Front Neurosci. (2015) 9. doi: 10.3389/fnins.2015.00041, PMID: 25729348 PMC4325937

[43] Ha M Kim VN. Regulation of microRNA biogenesis. Nat Rev Mol Cell Biol. (2014), 509–24. doi: 10.1038/nrm3838, PMID: 25027649

[44] Roberts TC. The microRNA machinery. In MicroRNA: Basic Science: Molecular Biology to Clinical Practice. Cham: Springer International Publishing. (2015), p. 15–30. doi: 10.1007/978-3-319-22380-3_2, PMID:

[45] Li Y Kowdley KV. MicroRNAs in common human diseases. Genomics Proteomics Bioinf. (2012), 246–53. doi: 10.1016/j.gpb.2012.07.005, PMID: 23200134 PMC3611977

[46] Fernández-Hernando C Ramírez CM Goedeke L Suárez Y. MicroRNAs in metabolic disease. Arterioscler Thromb Vasc Biol. (2013) 33:178–85. doi: 10.1161/ATVBAHA.112.300144, PMID: 23325474 PMC3740757

[47] Rottiers V Najafi-Shoushtari SH Kristo F Gurumurthy S Zhong L Li Y. Micrornas in metabolism and metabolic diseases. Cold Spring Harb Symp Quant Biol. (2011) 76:225–33. doi: 10.1101/sqb.2011.76.011049, PMID: 22156303 PMC3880782

[48] Young JI Nica Otero V Cerdá MG Falzone TSL Chan EC Low MJ. Authentic cell-specific and developmentally regulated expression of pro-opiomelanocortin genomic fragments in hypothalamic and hindbrain neurons of transgenic mice. The Journal of Neuroscience. (1998) 18(17):6631–40. doi: 10.1523/JNEUROSCI.18-17-06631.1998, PMID: 9712635 PMC6792967

[49] Diano S. New aspects of melanocortin signaling: A role for PRCP in α-MSH degradation. Front Neuroendocrinology. (2011), 70–83. doi: 10.1016/j.yfrne.2010.09.001, PMID: 20932857 PMC4766861

[50] Krieger DT Yamaguchi H Liotta AS. Human plasma ACTH, lipotropin, and endorphin. Adv Biochem Psychopharmacol. (1981) 28:541–56. 6259909

[51] Toda C Santoro A Kim JD Diano S. The annual review of physiology. Annu Rev Physiol. (2017) 79(1):209–36. doi: 10.1146/annurev-physiol-022516-034110, PMID: 28192062 PMC5669621

[52] Millington GWM. The role of proopiomelanocortin (POMC) neurones in feeding behaviour. Nutr Metab. (2007) 4(1):18. doi: 10.1186/1743-7075-4-18, PMID: 17764572 PMC2018708

[53] Faulkner LD Dowling AR Stuart RC Nillni EA Hill JW. Reduced melanocortin production causes sexual dysfunction in male mice with POMC neuronal insulin and leptin insensitivity. Endocrinol (United States). (2015) 156:1372–85. doi: 10.1210/en.2014-1788, PMID: 25590244 PMC4399313

[54] Wang W Guo DY Lin YJ Tao YX. Melanocortin regulation of inflammation. Front Endocrinol. (2019) 10. doi: 10.3389/fendo.2019.00683, PMID: 31649620 PMC6794349

[55] Qu N He Y Wang C Xu P Yang Y Cai X. A POMC-originated circuit regulates stress-induced hypophagia, depression, and anhedonia. Mol Psychiatry. (2020) 25:1006–21. doi: 10.1038/s41380-019-0506-1, PMID: 31485012 PMC7056580

[56] Krude H Biebermann H Luck W Horn R Brabant G Grüters A. Severe early-onset obesity, adrenal insufficiency and red hair pigmentation caused by POMC mutations in humans. Nat Genet. (1998) 19:155–7. doi: 10.1038/509, PMID: 9620771

[57] Farooqi IS Drop S Clements A Keogh JM Biernacka J Lowenbein S. Heterozygosity for a POMC-null mutation and increased obesity risk in humans. Diabetes. (2006) 55:2549–53. doi: 10.2337/db06-0214, PMID: 16936203

[58] Yaswen L Diehl N Brennan MB Hochgeschwender U. Obesity in the mouse model of pro-opiomelanocortin deficiency responds to peripheral melanocortin. Nat Med. (1999) 5:1066–70. doi: 10.1038/12506, PMID: 10470087

[59] Chhabra KH Adams JM Fagel B Lam DD Qi N Rubinstein M. Hypothalamic POMC deficiency improves glucose tolerance despite insulin resistance by increasing Glycosuria. Diabetes. (2016) 65:660–72. doi: 10.2337/db15-0804, PMID: 26467632 PMC4764146

[60] Shi C Lu Y Zhai G Huang J Shang G Lou Q. Hyperandrogenism in POMCa-deficient zebrafish enhances somatic growth without increasing adiposity. J Mol Cell Biol. (2020) 12:291–304. doi: 10.1093/jmcb/mjz053, PMID: 31237951 PMC7232124

[61] Yang Z Wong J Wang L Sun F Yue GH. pomc knockout increases growth in zebrafish. Aquaculture. (2023), 574. doi: 10.1016/j.aquaculture.2023.739707

[62] Fei F Sun SY Yao YX Wang X. Generation and phenotype analysis of zebrafish mutations of obesity-related genes lepr and mc4r. Sheng Li Xue Bao. (2017) 69:61–9., PMID: 28217809

[63] Cifani C Micioni Di Bonaventura MV Pucci M Giusepponi ME Romano A Di Francesco A. Regulation of hypothalamic neuropeptides gene expression in diet induced obesity resistant rats: Possible targets for obesity prediction? Front Neurosci. (2015) 9. doi: 10.3389/fnins.2015.00187, PMID: 26106286 PMC4458694

[64] Marco A Kisliouk T Tabachnik T Weller A Meiri N. DNA CpG methylation (5-methylcytosine) and its derivative (5-hydroxymethylcytosine) alter histone posttranslational modifications at the Pomc promoter, affecting the impact of perinatal diet on leanness and obesity of the offspring. Diabetes. (2016) 65:2258–67. doi: 10.2337/db15-1608, PMID: 27217481

[65] Kühnen P Handke D Waterland RA Hennig BJ Silver M Fulford AJ. Interindividual variation in DNA methylation at a putative POMC metastable epiallele is associated with obesity. Cell Metab. (2016) 24:502–9. doi: 10.1016/j.cmet.2016.08.001, PMID: 27568547

[66] Crujeiras AB Campion J Díaz-Lagares A Milagro FI Goyenechea E Abete I. Association of weight regain with specific methylation levels in the NPY and POMC promoters in leukocytes of obese men: A translational study. Regul Pept. (2013) 186:1–6. doi: 10.1016/j.regpep.2013.06.012, PMID: 23831408

[67] Ehrlich S Weiss D Burghardt R Infante-Duarte C Brockhaus S Muschler MA. Promoter specific DNA methylation and gene expression of POMC in acutely underweight and recovered patients with anorexia nervosa. J Psychiatr Res. (2010) 44:827–33. doi: 10.1016/j.jpsychires.2010.01.011, PMID: 20176366

[68] Wu Y Patchev AV Daniel G Almeida OFX Spengler D. Early-Life stress reduces dna methylation of the pomc gene in male mice. Endocrinology. (2014) 155:1751–62. doi: 10.1210/en.2013-1868, PMID: 24506071

[69] Rancourt RC Schellong K Tzschentke B Henrich W Plagemann A. DNA methylation and expression of proopiomelanocortin (POMC) gene in the hypothalamus of three-week-old chickens show sex-specific differences. FEBS Open Bio. (2018) 8:932–9. doi: 10.1002/feb4.2018.8.issue-6, PMID: 29928573 PMC5985994

[70] Sampey BP Vanhoose AM Winfield HM Freemerman AJ Muehlbauer MJ Fueger PT. Cafeteria diet is a robust model of human metabolic syndrome with liver and adipose inflammation: comparison to high-fat diet. Obesity. (2011) 19:1109–17. doi: 10.1038/oby.2011.18, PMID: 21331068 PMC3130193

[71] Lazzarino GP Acutain MF Canesini G Andreoli MF Ramos JG. Cafeteria diet induces progressive changes in hypothalamic mechanisms involved in food intake control at different feeding periods in female rats. Mol Cell Endocrinol. (2019) 498:110542. doi: 10.1016/j.mce.2019.110542, PMID: 31430504

[72] McFadden T Gaito N Carucci I Fletchall E Farrell K Jarome TJ. Controlling hypothalamic DNA methylation at the Pomc promoter does not regulate weight gain during the development of obesity. PloS One. (2023) 18(4):e0284286. doi: 10.1371/journal.pone.0284286, PMID: 37036864 PMC10085038

[73] Mahmood S Smiraglia DJ Srinivasan M Patel MS. Epigenetic changes in hypothalamic appetite regulatory genes may underlie the developmental programming for obesity in rat neonates subjected to a high-carbohydrate dietary modification. J Dev Orig Health Dis. (2013) 4:479–90. doi: 10.1017/S2040174413000238, PMID: 24924227

[74] Kokare DM Kyzar EJ Zhang H Sakharkar AJ Pandey SC. Adolescent alcohol exposure-induced changes in alpha-melanocyte stimulating hormone and neuropeptide y pathways via histone acetylation in the brain during adulthood. Int J Neuropsychopharmacol. (2017) 20:758–68. doi: 10.1093/ijnp/pyx041, PMID: 28575455 PMC5581492

[75] Bekdash RA Zhang C Sarkar DK. Gestational choline supplementation normalized fetal alcohol-induced alterations in histone modifications, DNA methylation, and proopiomelanocortin (POMC) gene expression in β-endorphin-producing POMC neurons of the hypothalamus. Alcohol Clin Exp Res. (2013) 37:1133–42. doi: 10.1111/acer.2013.37.issue-7, PMID: 23413810 PMC3659188

[76] Derghal A Djelloul M Airault C Pierre C Dallaporta M Troadec JD. Leptin is required for hypothalamic regulation of miRNA stargeting POMC 3′UTR. Front Cell Neurosci. (2015) 9. doi: 10.3389/fncel.2015.00172, PMID: 25999818 PMC4422035

[77] Gangisetty O Chaudhary S Tarale P Cabrera MA Sarkar DK. miRNA-383 and miRNA-384 suppress proopiomelanocortin gene expression in the hypothalamus: effects of early life ethanol exposure. Neuroendocrinology. (2023) 113:844–58. doi: 10.1159/000530289, PMID: 36948162 PMC10389800

[78] Zhang D Yamaguchi S Zhang X Yang B Kurooka N Sugawara R. Upregulation of mir342 in diet-induced obesity mouse and the hypothalamic appetite control. Front Endocrinol (Lausanne). (2021) 12. doi: 10.3389/fendo.2021.727915, PMID: 34526970 PMC8437242

[79] Ma Y Murgia N Liu Y Li Z Sirakawin C Konovalov R. Neuronal miR-29a protects from obesity in adult mice. Mol Metab. (2022) 61:101507. doi: 10.1016/j.molmet.2022.101507, PMID: 35490865 PMC9114687

[80] Zhang N Lin JK Chen J Liu XF Liu JL Luo HS. MicroRNA 375 mediates the signaling pathway of corticotropin-releasing factor (CRF) regulating pro-opiomelanocortin (POMC) Expression by targeting Mitogen-activated protein Kinase 8. J Biol Chem. (2013) 288:10361–73. doi: 10.1074/jbc.M112.425504, PMID: 23430746 PMC3624419

[81] Lechner L Opitz R Silver MJ Krabusch PM Prentice AM Field MS. Early-set POMC methylation variability is accompanied by increased risk for obesity and is addressable by MC4R agonist treatment. Sci Transl Med. (2023). doi: 10.1126/scitranslmed.adg1659, PMID: 37467315

[82] Schroeder JW Conneely KN Cubells JC Kilaru V Jeffrey Newport D Knight BT. Neonatal DNA methylation patterns associate with gestational age. Epigenetics. (2011) 6:1498–504. doi: 10.4161/epi.6.12.18296, PMID: 22139580 PMC3256334

[83] Zheng J Xiao X Zhang Q Yu M Xu J Wang Z. Maternal and post-weaning high-fat, high-sucrose diet modulates glucose homeostasis and hypothalamic POMC promoter methylation in mouse offspring. Metab Brain Dis. (2015) 30:1129–37. doi: 10.1007/s11011-015-9678-9, PMID: 25936720

[84] Yang YK Thompson DA Dickinson CJ Wilken J Barsh GS Kent SB. Characterization of agouti-related protein binding to melanocortin receptors. Molecular Endocrinology. (1999) 13(1):148–55. doi: 10.1210/mend.13.1.0223, PMID: 9892020

[85] Nijenhuis WAJ Oosterom J Adan RAH. AgRP(83 - 132) acts as an inverse agonist on the human-melanocortin-4 receptor. Molecular Endocrinology. (2001) 15(1):164–71. doi: 10.1210/mend.15.1.0578, PMID: 11145747

[86] Ming Fong T Mao C MacNeil T Kalyani R Smith T Weinberg D. ART (Protein product of agouti-related transcript) as an antagonist of MC - 3 and MC - 4 receptors. (1997) 237:., PMID: 9299416 10.1006/bbrc.1997.7200

[87] Krashes MJ Koda S Ye CP Rogan SC Adams AC Cusher DS. Rapid, reversible activation of AgRP neurons drives feeding behavior in mice. J Clin Invest. (2011) 121:1424–8. doi: 10.1172/JCI46229, PMID: 21364278 PMC3069789

[88] Morrison CD Xi X White CL Ye J Martin RJ. Amino acids inhibit Agrp gene expression via an mTOR-dependent mechanism. Am J Physiol Endocrinol Metab. (2007) 293:165–71. doi: 10.1152/ajpendo.00675.2006, PMID: 17374702 PMC2596875

[89] Barnes MJ Argyropoulos G Bray GA. Preference for a high fat diet, but not hyperphagia following activation of mu opioid receptors is blocked in AgRP knockout mice. Brain Res. (2010) 1317:100–7. doi: 10.1016/j.brainres.2009.12.051, PMID: 20051234 PMC2829843

[90] Thomas MA Tran V Ryu V Xue B Bartness TJ. AgRP knockdown blocks long-term appetitive, but not consummatory, feeding behaviors in Siberian hamsters. Physiol Behav. (2018), 61–70. doi: 10.1016/j.physbeh.2017.10.008, PMID: 29031552 PMC5897226

[91] Lazzarino GP Andreoli MF Rossetti MF Stoker C Tschopp MV Luque EH. Cafeteria diet differentially alters the expression of feeding-related genes through DNA methylation mechanisms in individual hypothalamic nuclei. Mol Cell Endocrinol. (2017) 450:113–25. doi: 10.1016/j.mce.2017.05.005, PMID: 28479374

[92] Coupé B Amarger V Grit I Benani A Parnet. Nutritional programming affects hypothalamic organization and early response to leptin. Endocrinology. (2010) 151:702–13. doi: 10.1210/en.2009-0893, PMID: 20016030

[93] Shin BC Dai Y Thamotharan M Gibson LC Devaskar SU. Pre- and postnatal calorie restriction perturbs early hypothalamic neuropeptide and energy balance. J Neurosci Res. (2012) 90:1169–82. doi: 10.1002/jnr.23013, PMID: 22388752 PMC4208917

[94] Schellong K Melchior K Ziska T Henrich W Rancourt RC Plagemann A. Sex-specific epigenetic alterations of the hypothalamic Agrp-Pomc system do not explain ‘diabesity’ in the offspring of high-fat diet (HFD) overfed maternal rats. J Nutr Biochem. (2020) 75:108257. doi: 10.1016/j.jnutbio.2019.108257, PMID: 31710935

[95] Rapps K Kisliouk T Marco A Weller A Meiri N. Dieting reverses histone methylation and hypothalamic AgRP regulation in obese rats. Front Endocrinol (Lausanne). (2023) 14. doi: 10.3389/fendo.2023.1121829, PMID: 36817590 PMC9930686

[96] Desai M Han G Ross MG. Programmed hyperphagia in offspring of obese dams: Altered expression of hypothalamic nutrient sensors, neurogenic factors and epigenetic modulators. Appetite. (2016) 99:193–9. doi: 10.1016/j.appet.2016.01.023, PMID: 26785315 PMC4813304

[97] Przybył BJ Szlis M Wójcik-Gładysz A. Brain-Derived Neurotrophic Factor Affects mRNA and miRNA Expression of the Appetite Regulating Centre in the Sheep Arcuate Nucleus. Ann Anim Science. (2020) 20:853–69. doi: 10.2478/aoas-2020-0015

[98] Lin S Boey D Herzog H. NPY and Y receptors: Lessons from transgenic and knockout models. Neuropeptides. (2004), 189–200. doi: 10.1016/j.npep.2004.05.005, PMID: 15337371

[99] Mercer RE Chee MJS Colmers WF. The role of NPY in hypothalamic mediated food intake. Front Neuroendocrinology. (2011), 398–415. doi: 10.1016/j.yfrne.2011.06.001, PMID: 21726573

[100] Horsnell H Baldock PA. Osteoblastic actions of the neuropeptide Y system to regulate bone and energy homeostasis. Curr Osteoporosis Rep. (2016), 26–31. doi: 10.1007/s11914-016-0300-9, PMID: 26872458

[101] Engström Ruud L Pereira MMA de Solis AJ Fenselau H Brüning JC. NPY mediates the rapid feeding and glucose metabolism regulatory functions of AgRP neurons. Nat Commun. (2020) 11:442. doi: 10.1038/s41467-020-14291-3, PMID: 31974377 PMC6978463

[102] Erickson JC Clegg KE Palmiter RD. Sensitivity to leptin and susceptibility to seizures of mice lacking neuropeptide Y. Nature. (1996) 381:415–8. doi: 10.1038/381415a0, PMID: 8632796

[103] Cabrera Zapata LE Cambiasso MJ Arevalo MA. Epigenetic modifier Kdm6a/Utx controls the specification of hypothalamic neuronal subtypes in a sex-dependent manner. Front Cell Dev Biol. (2022) 10. doi: 10.3389/fcell.2022.937875, PMID: 36268511 PMC9577230

[104] Balasubramanian N Sagarkar S Jadhav M Shahi N Sirmaur R Sakharkar AJ. Role for histone deacetylation in traumatic brain injury-induced deficits in neuropeptide y in arcuate nucleus: possible implications in feeding behavior. Neuroendocrinology. (2021) 111:1187–200. doi: 10.1159/000513638, PMID: 33291119

[105] Krunic A Loganathan N Nkechika V Belsham DD. Phenylbutyric acid robustly increases Npy mRNA expression in hypothalamic neurons by increasing H3K9/14 acetylation at the Npy promoter. Biochem Biophys Res Commun. (2023) 658:18–26. doi: 10.1016/j.bbrc.2023.03.031, PMID: 37011479

[106] Croizier S Park S Maillard J Bouret SG. Central Dicer-miR-103/107 controls developmental switch of POMC progenitors into NPY neurons and impacts glucose homeostasis. Elife. (2018) 7. doi: 10.7554/eLife.40429, PMID: 30311908 PMC6203430

[107] Feng X Ding Y Zhou M Song N Ding Y. Integrative Analysis of Exosomal miR-452 and miR-4713 Downregulating NPY1R for the Prevention of Childhood Obesity. Dis Markers. (2022) 2022:1–12. doi: 10.1155/2022/2843353, PMID: 35401881 PMC8986441

[108] Tartaglia LA Dembski M Weng X Deng N Culpepper J Devos R. Identification and expression cloning of a leptin receptor, OB-R. Cell. (1995). doi: 10.1016/0092-8674(95)90151-5, PMID: 8548812

[109] Peelman F Waelput W Iserentant H Lavens D Eyckerman S Zabeau L. Leptin: Linking adipocyte metabolism with cardiovascular and autoimmune diseases. Prog Lipid Res. (2004), 283–301. doi: 10.1016/j.plipres.2004.03.001, PMID: 15234549

[110] Tartaglia LA. The leptin receptor. J Biol Chem. (1997), 6093–6. doi: 10.1074/jbc.272.10.6093, PMID: 9102398

[111] Bates SH Myers MG. The role of leptin receptor signaling in feeding and neuroendocrine function. Trends Endocrinol Metab. (2003), 447–52. doi: 10.1016/j.tem.2003.10.003, PMID: 14643059

[112] Cohen P Zhao C Cai X Montez JM Rohani SC Feinstein P. Selective deletion of leptin receptor in neurons leads to obesity. J Clin Invest. (2001) 108:1113–21. doi: 10.1172/JCI200113914, PMID: 11602618 PMC209535

[113] Nunziata A Funcke JB Borck G Von Schnurbein J Brandt S Lennerz B. Functional and phenotypic characteristics of human leptin receptor mutations. J Endocr Soc. (2019) 3:27–41. doi: 10.1210/js.2018-00123, PMID: 30560226 PMC6293235

[114] Childs GV Akhter N Haney A Syed M Odle A Cozart M. The somatotrope as a metabolic sensor: Deletion of leptin receptors causes obesity. Endocrinology. (2011) 152:69–81. doi: 10.1210/en.2010-0498, PMID: 21084451 PMC3033057

[115] Zhang Q Xiao X Zheng J Li M Yu M Ping F. Maternal inulin alleviates high-fat diet-induced lipid disorder in offspring by epigenetically modulating hypothalamus feeding circuit-related genes. Food Funct. (2023). doi: 10.1039/D3FO02223D, PMID: 38044717

[116] Fan C Liu X Shen W Deckelbaum RJ Qi K. The regulation of leptin, leptin receptor and pro-opiomelanocortin expression by N - 3 PUFAs in diet-induced obese mice is not related to the methylation of their promoters. Nutr Metab (Lond). (2011) 8(1):31. doi: 10.1186/1743-7075-8-31, PMID: 21609458 PMC3117679

[117] Batury VL Walton E Tam F Wronski ML Buchholz V Frieling H. DNA methylation of ghrelin and leptin receptors in underweight and recovered patients with anorexia nervosa. J Psychiatr Res. (2020) 131:271–8. doi: 10.1016/j.jpsychires.2020.08.026, PMID: 33091847

[118] Palou M Picó C McKay JA Sánchez J Priego T Mathers JC. Protective effects of leptin during the suckling period against later obesity may be associated with changes in promoter methylation of the hypothalamic pro-opiomelanocortin gene. Br J Nutr. (2011), 769–78. doi: 10.1017/S0007114511000973, PMID: 21554805

[119] Kim MH Li Y Zheng Q Jiang L Myers MG Wu WS. LepRb+ cell–specific deletion of Slug mitigates obesity and nonalcoholic fatty liver disease in mice. J Clin Invest. (2023) 133(4). doi: 10.1172/JCI156722, PMID: 36512408 PMC9927931

[120] Crépin D Benomar Y Riffault L Amine H Gertler A Taouis M. The over-expression of miR-200a in the hypothalamus of ob/ob mice is linked to leptin and insulin signaling impairment. Mol Cell Endocrinol. (2014) 384:1–11. doi: 10.1016/j.mce.2013.12.016, PMID: 24394757

[121] Schulingkamp RJ Pagano TC Hung D Raffa RB. Insulin receptors and insulin action in the brain: review and clinical implications. Neurosci Biobehav Rev. (2000) 24(8):855–72. doi: 10.1016/S0149-7634(00)00040-3, PMID: 11118610

[122] Werther GA Hogg A Oldfield BJ Mckinley MJ Figdor R Allen AM. Localization and characterization of insulin receptors in rat brain and pituitary gland using *in vitro* autoradiography and computerized densitometry*. Endocrinology. (1987) 121:1562–70. doi: 10.1210/endo-121-4-1562, PMID: 3653038

[123] Folli F Ghidella S Bonfanti L Kahn CR Merighi A. The early intracellular signaling pathway for the insulin/insulin-like growth factor receptor family in the mammalian central nervous system. Mol Neurobiol. (1996) 13:155–83. doi: 10.1007/BF02740639, PMID: 8938649

[124] Mosthaf L Grako K Dull TJ Coussens L Ullrich A McClain DA. Functionally distinct insulin receptors generated by tissue-specific alternative splicing. EMBO J. (1990) 9:2409–13. doi: 10.1002/j.1460-2075.1990.tb07416.x, PMID: 2369896 PMC552265

[125] Frasca F Pandini G Scalia P Sciacca L Mineo R Costantino A. a newly recognized, high-affinity insulin-like growth factor II receptor in fetal and cancer cells. Mol Cell Biol. (1999) 19:3278–88. doi: 10.1128/MCB.19.5.3278, PMID: 10207053 PMC84122

[126] Dodd GT Tiganis T. Insulin action in the brain: Roles in energy and glucose homeostasis. J Neuroendocrinol. (2017) 29(10). doi: 10.1111/jne.2017.29.issue-10, PMID: 28758251

[127] Masaki T Chiba S Noguchi H Yasuda T Tobe K Suzuki R. Obesity in insulin receptor substrate-2–deficient mice: disrupted control of arcuate nucleus neuropeptides. Obes Res. (2004) 12:878–85. doi: 10.1038/oby.2004.106, PMID: 15166310

[128] Obici S Feng Z Karkanias G Baskin DG Rossetti L. Decreasing hypothalamic insulin receptors causes hyperphagia and insulin resistance in rats. Nat Neurosci. (2002) 5:566–72. doi: 10.1038/nn0602-861, PMID: 12021765

[129] Paranjape SA Chan O Zhu W Horblitt AM Grillo CA Wilson S. Chronic reduction of insulin receptors in the ventromedial hypothalamus produces glucose intolerance and islet dysfunction in the absence of weight gain. Am J Physiol Endocrinol Metab. (2011) 301:978–83. doi: 10.1152/ajpendo.00304.2011, PMID: 21828334 PMC3774165

[130] Plagemann A Roepke K Harder T Brunn M Harder A Wittrock-Staar M. Epigenetic malprogramming of the insulin receptor promoter due to developmental overfeeding. J Perinat Med. (2010) 38:393–400. doi: 10.1515/jpm.2010.051, PMID: 20443665

[131] Schellong K Melchior K Ziska T Ott R Henrich W Rancourt RC. Hypothalamic insulin receptor expression and DNA promoter methylation are sex-specifically altered in adult offspring of high-fat diet (HFD)-overfed mother rats. J Nutr Biochem. (2019) 67:28–35. doi: 10.1016/j.jnutbio.2019.01.014, PMID: 30849557

[132] Pandur E Szabó I Hormay E Pap R Almási A Sipos K. Alterations of the expression levels of glucose, inflammation, and iron metabolism related miRNAs and their target genes in the hypothalamus of STZ-induced rat diabetes model. Diabetol Metab Syndr. (2022) 14(1):147. doi: 10.1186/s13098-022-00919-5, PMID: 36210435 PMC9549668

[133] Menyhért J Wittmann G Lechan RM Keller E Liposits Z Fekete C. Cocaine- and amphetamine-regulated transcript (CART) is colocalized with the orexigenic neuropeptide Y and agouti-related protein and absent from the anorexigenic α-melanocyte-stimulating hormone neurons in the infundibular nucleus of the human hypothalamus. Endocrinology. (2007) 148:4276–81. doi: 10.1210/en.2007-0390, PMID: 17525122

[134] Wierup N Richards WG Bannon AW Kuhar MJ Ahrén B Sundler F. CART knock out mice have impaired insulin secretion and glucose intolerance, altered beta cell morphology and increased body weight. Regul Pept. (2005) 129:203–11. doi: 10.1016/j.regpep.2005.02.016, PMID: 15927717

[135] Asnicar MA Smith DP Yang DD Heiman ML Fox N Chen YF. Absence of cocaine- and amphetamine-regulated transcript results in obesity in mice fed a high caloric diet. Endocrinology. (2001) 142:4394–400. doi: 10.1210/endo.142.10.8416, PMID: 11564703

[136] Gibson LC Shin BC Dai Y Freije W Kositamongkol S Cho J. Early leptin intervention reverses perturbed energy balance regulating hypothalamic neuropeptides in the pre- and postnatal calorie-restricted female rat offspring. J Neurosci Res. (2015) 93:902–12. doi: 10.1002/jnr.23560, PMID: 25639584 PMC4533910

[137] Osei-Hyiaman D Harvey-White J Bátkai S Kunos G. The role of the endocannabinoid system in the control of energy homeostasis. Int J Obes. (2006), S33–8. doi: 10.1038/sj.ijo.0803276, PMID: 16570103

[138] Cruz-Martínez AM Tejas-Juárez JG Mancilla-Díaz JM Florán-Garduño B López-Alonso VE Escartín-Pérez RE. CB1 receptors in the paraventricular nucleus of the hypothalamus modulate the release of 5-HT and GABA to stimulate food intake in rats. Eur Neuropsychopharmacol. (2018) 28:1247–59. doi: 10.1016/j.euroneuro.2018.08.002, PMID: 30217553

[139] Quarta C Bellocchio L Mancini G Mazza R Cervino C Braulke LJ. CB1 signaling in forebrain and sympathetic neurons is a key determinant of endocannabinoid actions on energy balance. Cell Metab. (2010) 11:273–85. doi: 10.1016/j.cmet.2010.02.015, PMID: 20374960

[140] Almeida MM Dias-Rocha CP Reis-Gomes CF Wang H Atella GC Cordeiro A. Maternal high-fat diet impairs leptin signaling and up-regulates type-1 cannabinoid receptor with sex-specific epigenetic changes in the hypothalamus of newborn rats. Psychoneuroendocrinology. (2019) 103:306–15. doi: 10.1016/j.psyneuen.2019.02.004, PMID: 30776574

[141] Basu R Flak JN. Hypothalamic neural circuits regulating energy expenditure. (2025), 79–124. doi: 10.1016/bs.vh.2024.07.004, PMID: 39864947 PMC12007011

[142] Sarrafha L Neavin DR Parfitt GM Kruglikov IA Whitney K Reyes R. Novel human pluripotent stem cell-derived hypothalamus organoids demonstrate cellular diversity. iScience. (2023) 26(9):107525. doi: 10.1016/j.isci.2023.107525, PMID: 37646018 PMC10460991

[143] Fu T Kemper JK. MicroRNA-34a and impaired FGF19/21 signaling in obesity. Vitamins Hormones. Academic Press Inc.; (2016), 175–96. doi: 10.1016/bs.vh.2016.02.002, PMID: PMC4977581 27125742

[144] Lavery CA Kurowska-Stolarska M Holmes WM Donnelly I Caslake M Collier A. miR-34a–/– mice are susceptible to diet-induced obesity. Obesity. (2016) 24:1741–51. doi: 10.1002/oby.21561, PMID: 27377585 PMC4979678

[145] Briones-Espinoza MJ Cortés-García JD Vega-Cárdenas M Uresti-Rivera EU Gómez-Otero A López-López N. Decreased levels and activity of Sirt1 are modulated by increased miR-34a expression in adipose tissue mononuclear cells from subjects with overweight and obesity: A pilot study. Diabetes Metab Syndrome: Clin Res Rev. (2020) 14:1347–54. doi: 10.1016/j.dsx.2020.07.014, PMID: 32755834

[146] Di Bello E Noce B Fioravanti R Mai A. Current HDAC inhibitors in clinical trials. Chimia (Aarau). (2022) 76:448–53. doi: 10.2533/chimia.2022.448, PMID: 38069716

[147] West AC Johnstone RW. New and emerging HDAC inhibitors for cancer treatment. J Clin Invest. (2014), 30–9. doi: 10.1172/JCI69738, PMID: 24382387 PMC3871231

[148] Hontecillas-Prieto L Flores-Campos R Silver A de Álava E Hajji N García-Domínguez DJ. Synergistic enhancement of cancer therapy using HDAC inhibitors: opportunity for clinical trials. Front Genet. (2020) 11. doi: 10.3389/fgene.2020.578011, PMID: 33024443 PMC7516260

[149] Keller K Jung M. Histone deacetylase (HDAC) inhibitors in recent clinical trials for cancer therapy. Epigenetic Ther Cancer. (2014), 227–55. doi: 10.2533/chimia.2022.448, PMID: 21258646 PMC3020651

