# Supplementary material for: Epigenetic modification of hypothalamic neuropeptides and metabolic hormone receptors in metabolic health
Source: Front Endocrinol (Lausanne). 2025 Sep 17;16:1645474. doi: 10.3389/fendo.2025.1645474 (PMC12483883; doi:10.3389/fendo.2025.1645474)
Supplement: Supplementary file 1 [file DataSheet1.pdf]

Table 1: Epigenetic modifications of *Pomc*

Status	Model	Modification	Brief Phenotype	Reference
DNA Methylation	Rats on HFD(45% fat)	↓ DNA methylation at CpG sites 1, 2, 6, 7	Diet-resistant rats showed ↑ Pomc expression and ↓ promoter methylation	(63)
	Rat (HFD-fed from post-weaning to adulthood)	DNA hypermethylation in the *Pomc* promoter	↓ *Pomc* expression; ↑ body weight compared to control	(64)
	Human (postmortem hypothalamic tissue)	↑ methylation of *Pomc* promoter at CpG island (intron-exon 3 region)	↑ Methylation in MSH neurons correlates with ↑ BMI, suggesting impaired satiety signaling and obesity development	(65)
	Human (Clinical study)	DNA methylation (CpG 10 &11)	↑ Baseline methylation at CpG sites 10 & 11 in weight regainers compared to non-regainers after a hypocaloric diet suggests epigenetic differences may predict weight regain.	(66)
	Human (PBMCs) from healthy women, underweight anorexia nervosa patients, and weight-recovered patients	DNA methylation at specific CpG sites	↑ *Pomc* expression in underweight anorexia nervosa patients; expression linked to site-specific CpG methylation rather than global methylation differences.	(67)
	Rodent pups exposed to early life stress (maternal separation: 3 hrs/day for 10 days)	↓ DNA methylation	↑ *Pomc* mRNA in the pituitary gland	(68)
	Rats on CAF	Demethylation of *Pomc* promoter	↓ *Pomc* methylation after CAF diet exposure, but insufficient to prevent ↑ food intake and body weight gain, implying other orexigenic mechanisms are involved.	(71)
	Offspring of ethanol-exposed dams	↑ DNA methylation (via ↑ DNMT1)	↓ *Pomc* mRNA expression in offspring	(85)
	Human embryonic stem cells (naive, primed H1, capacitated)	DNA methylation (CpG 1–7)	Naive hESCs showed ↓ methylation; capacitated cells showed ↑ methylation at progenitor and neuronal stages. There was an inverse relationship between DNA methylation and *Pomc* gene expression during hypothalamic differentiation.	(81)
	Offspring of dams fed a high-fat, high-sucrose diet during pregnancy	Hypomethylation of *Pomc* promoter in the hypothalamus	↑ *Pomc* expression and metabolic dysfunction in adulthood	(83)

Histone modifications	Pups fed a high-carb diet	↓ H3K9ac at *Pomc* promoter	↓ *Pomc* expression; ↑ body weight	(73)
	Adolescent alcohol exposed rats	↑ H3K9/14 at promoter	↑ *Pomc* mRNA expression persists into adulthood	(74)
	Rat offspring (prenatal ethanol exposure)	↓ H3K4me3 (activation mark), ↑ H3K9me2 (repressive mark)	↓ *Pomc* mRNA levels; potential disruption in stress axis and metabolic control	(75)
miRNAs modifications	Mouse (prenatal alcohol exposure, PND 2–6)	↑miR-383 and miR-384	↓ *Pomc* expression in the mediobasal hypothalamus at PND 6 and 60 indicates altered metabolic regulation, possibly due to prenatal alcohol exposure	(77)
	Mouse	High-fat, high-sucrose diet + genetic knockdown of miR-342	↑ *Pomc* expression, ↓ food intake, and ↓ body weight suggest a role of miR-342 in obesity progression	(78)
	Female mice with *Pomc* neuron-specific miR-29a deletion	Conditional knockout in *Pomc* neurons	Hyperphagia, ↓ energy expenditure, and obesity	(79)
	Loss or gain function	Inhibition and upregulation of miR-375	Inhibition of miR-375 led to ↑ *Pomc* expression, while upregulation of it led to ↓ *Pomc* expression levels	(80)

Table 2: Epigenetic modifications of *AgRP*

Status	Model	Modification	Brief Phenotype	Reference
DNA Methylation	Rat pups (offspring of calorie-restricted dams)	↑ DNA methylation at three CpG sites (75%–96%) in the promoter (no CpG island)	↑ *AgRP* expression despite ↓ weight gain, suggesting disrupted *AgRP* signaling during undernutrition.	(93)
	Maternal HFD-exposed offspring (male)	↑ DNA methylation at four CpG sites	↓ *AgRP* expression in male offspring: sex-specific epigenetic regulation	(94)
Histone modifications	Caloric restriction following HFD-induced obesity	↓ H3K9me2 (repressive histone methylation) at the promoter	↑ Hunger signaling as an adaptive response to restore energy homeostasis	(95)
	Male offspring exposed to maternal HFD (60% kcal from fat)	↑ LSD1 (lysine-specific histone demethylase 1), ↓ HDAC1 (histone deacetylase 1); histone demethylation and acetylation	↑ *AgRP* mRNA expression at 6 months, indicating increased appetite drive, suggests sex-specific epigenetic regulation of *AgRP* in response to maternal HFD	(96)
miRNAs modifications	Sheep injected with BDNF	↑ miRNA-33a-5p and miRNA-33b-5p, and ↓ miRNA-377-3p and miRNA-214-3p levels,	↑ *AgRP* expression suggests miRNA-mediated regulation of hypothalamic appetite signals.	(97)

Table 3: Epigenetic modifications of *Npy*

Status	Model	Modification	Brief Phenotype	Reference
DNA Methylation	Rats fed CAF	↓ DNA methylation	↑ *Npy* mRNA expression; associated with overeating and weight gain	(71)
	Diet-resistant vs. diet-induced obese rats	↑ DNA methylation at the 5th CpG site of the *Npy* promoter	↓ *Npy* expression, associated with lower food intake and resistance to weight gain	(63)
	Obese human males	DNA hypomethylation at CpG sites 4 and 8	↓ *Npy* expression, weight regainers showed lower methylation at baseline and after diet, compared to non-regainers	(66)
	Offspring of dams fed a high-carbohydrate diet	↓ DNA methylation at the *Npy* promoter	↑ *Npy* expression due to hypomethylation; hyperphagia, ↑ weight gain	(73)
Histone modifications	Rats (high-carb diet)	↑ H3K9 acetylation at the promoter	↑ *Npy* expression contributing to obesity	(73)
	Mouse hypothalamic neurons	Histone modification (trimethylation at H3K27)	Greater transcriptional suppression of Npy in males compared to females	(103)
	Mouse hypothalamic neurons	siRNA knockdown of Kdm6a (H3K27 demethylase)	Enhanced Npy repression in females; no significant change in males	(103)
	Traumatic Brain Injury (TBI) model	↓ H3K9ac at *Npy* promoter	↓ Food intake due to decreased *Npy* expression in the arcuate nucleus	(104)
	mHypoA-59 cells	↑ H3K9/14 acetylation at *Npy* promoter (via phenyl butyric acid, an HDAC inhibitor and ER stress inhibitor)	↑ *Npy* expression, linked to histone acetylation and the development of obesity	(105)
miRNAs modifications	Mouse (miR-342^{-}/$^{-}$)	Genetic deletion of miR-342	↓ activation of *Npy* in response to a high-fat, high-sugar diet; protected from obesity	(107)
	Human (obese children)	Predicted regulation by miR-4713 and miR-452 (using miRWalk2.0)	↑ Expression of *Npy*1*r* is associated with obesity	(108)

Table 4: Epigenetic modifications of *LepRb*

Status	Model	Modification	Brief Phenotype	References
DNA Methylation	Offspring of maternal high-fat diet (HFD) mice	↑ DNA methylation (global and promoter-specific)	↑ body weight gain and ↓ LepRb expression in offspring	(116)
Histone modifications	HFD-fed mice	↑ H3K27me2/3 at the LepRb promoter (via Slug TF activity)	↓ LepRb expression in the hypothalamus, potentially contributing to leptin resistance and obesity	(120)
miRNAs modifications	Hypothalamic miR-200 inhibition model	miR-200 inhibition	↓ food intake, and ↓ body weight	(121)

Table 5: Epigenetic modifications of *InsR*

Status	Model	Modification	Brief Phenotype	References
DNA Methylation	Neonatal overfed rats	↑ DNA methylation (CpG island of promoter)	Rapid weight gain; development of metabolic syndrome	(129)
	Maternal High-Fat Diet (HFD) mouse model	↑ DNA methylation	Hyperleptinemia, hyperinsulinemia, impaired glucose tolerance, insulin resistance, and obesity in male offspring	(130)
miRNAs modification	Diabetic rats	↑ miR-194-5p and miR-200a-3p	↓ InsR protein levels, impaired insulin signaling	(131)
	Diabetic rats	↓ miR-194-5p and miR-200a-3p	↑ InsR protein levels, enhanced insulin signaling	(131)
	db/db mice	↓ miR-200	↑ *Insr* expression, suppressed appetite, ↓ food intake, and ↓body weight	(120)
